# The Use of Risk Scores for Thromboprophylaxis in Medically Ill Patients—Rationale and Design of the RICO trial

**DOI:** 10.1055/a-2209-4708

**Published:** 2024-01-12

**Authors:** Francesco Dentali, Mauro Campanini, Aldo Bonaventura, Luca Fontanella, Francesca Zuretti, Luca Tavecchia, Nicola Mumoli, Paola Gnerre, Francesco Ventrella, Michela Giustozzi, Antonella Valerio, Andrea Fontanella

**Affiliations:** 1 Division of Internal Medicine, Medical Center, Ospedale di Circolo & Fondazion Macchi, ASST Sette Laghi, Varese, Italy; 2 Department of Medicine and Surgery, Insubria University, Varese, Italy; 3 Department of Internal Medicine, Hospital “Maggiore della Carità,” Novara, Italy; 4 Department of Medicine, Ospedale Buonconsiglio Fatebenefratelli di Napoli, Naples, Italy; 5 Department of Internal Medicine, Azienda Socio Sanitaria Territoriale (ASST) Ovest Milanese, Magenta, Italy; 6 Internal Medicine, “San Paolo” Hospital, Savona, Italy; 7 Department of Internal Medicine, Hospital “G. Tatarella”—ASL-FG, Cerignola, Italy; 8 Internal Vascular and Emergency Medicine—Stroke Unit, University of Perugia, Perugia, Italy; 9 Federazione delle Associazioni dei Dirigenti Ospedalieri Internisti (FADOI) Research Center, Milan, Italy

**Keywords:** medical patients, venous thromboembolism, RAMs, major bleeding, thromboprophylaxis

## Abstract

**Background** Venous thromboembolism (VTE) in hospitalized medically ill patients is a significant cause of morbidity and mortality. Guidelines suggest that VTE and bleeding risk assessment models (RAMs) should be integrated into the clinical decision-making process on thromboprophylaxis. However, poor evidence is available comparing the use of a RAM versus clinical judgement in evaluating VTE and bleeding occurrence.

**Methods** Reducing Important Clinical Outcomes in hospitalized medical ill patients (RICO) is a multicenter, cluster-randomized, controlled clinical trial (ClinicalTrials.gov Identifier: NCT04267718). Acutely ill patients hospitalized in Internal Medicine wards are randomized to the use of RAMs—namely the Padua Prediction Score and the International Medical Prevention Registry on Venous Thromboembolism Bleeding Score—or to clinical judgement. The primary study outcome is a composite of symptomatic objectively confirmed VTE and major bleeding at 90-day follow-up. Secondary endpoints include the evaluation of clinical outcomes at hospital discharge and the assessment of VTE prophylaxis prescription during the study period. In order to demonstrate a 50% reduction in the primary outcome in the experimental group and assuming an incidence of the primary outcome of 3.5% in the control group at 90-day; 2,844 patients across 32 centers will be included in the study.

**Discussion** The RICO trial is a randomized study of clinical management assessing the role of RAMs in hospitalized medical ill patients with the aim of reducing VTE and bleeding occurrence. The study has the potential to improve clinical practice since VTE still represents an important cause of morbidity and mortality in this setting.

## Introduction

Venous thromboembolism (VTE) in hospitalized patients with a medical illness is a significant cause of morbidity and mortality. 1 Recent European estimates report an annual incidence of VTE ranging from 104 to 183 per 100,000 person-years. 2 VTE is also associated with reduced survival. 2 The rationale for thromboprophylaxis in hospitalized, medically ill patients is supported by a wealth of evidence showing decreased pulmonary embolism and symptomatic deep vein thrombosis rates without significant increases in major bleedings. 3 4 In spite of this, thromboprophylaxis is generally underprescribed 1 as only 40 to 75% of eligible patients receive VTE prophylaxis. 5 6 7 Reasons accounting for suboptimal prescription of thromboprophylaxis may derive from an underestimation of the risk of VTE or from a risk–benefit evaluation, in terms of thrombotic and bleeding risk, that is sometimes challenging in patients hospitalized in Internal Medicine (IM) wards.

Most recent guidelines suggest that VTE and bleeding risk assessment models (RAMs) should be integrated into the clinical decision-making process. 4 To date, a number of RAMs evaluating VTE risk in hospitalized patients is available, as summarized in Table 1 . 8 9 10 11 12 13 14 15 16 17 18 19 20 The Padua Prediction Score and the International Medical Prevention Registry on Venous Thromboembolism (IMPROVE) VTE score are considered the best available RAMs to evaluate the risk for VTE in hospitalized, medically ill patients 15 18 ( Table 1 ). Padua Prediction Score includes a number of differently weighed conditions known to increase VTE risk, with a final score ≥4 suggesting a high risk for VTE. 8 The IMPROVE Bleeding Score was developed and validated to assess the risk of bleeding in a population of hospitalized, medically ill patients. 11 Among these patients, an IMPROVE Bleeding Score ≥ 7 conferred an increased risk of bleeding. 11 In a large single-center study (1,761 patients) focused on in-hospital medical patients, more than three-fourths of the included patients were at high VTE risk and almost 90% of them were at low bleeding risk. 10

**Table 1:** Main risk assessment models for venous thromboembolism prediction in hospitalized, medically ill patients

RAM	Authors	Type of study	Reference
*VTE RAM*			
Geneva Risk Score	Chopard et al	Retrospective cohort study (for derivation)	10
	Nendaz et al	Prospective cohort study (for validation)	14
Simplified Geneva Risk Score	Blondon et al	Prospective cohort study (for validation)	9
Padua Prediction Score	Barbar et al.	Prospective cohort study	8
	Vardi et al	Prospective cohort study (for validation)	18
IMPROVE VTE	Spyropoulos et al	Prospective cohort study	17
	Rosenberg et al	Case–control study (for validation)	15
	Mahan et al	Retrospective cohort study (for validation)	34
	Greene et al	Retrospective, cohort study (for validation)	35
Kucher RAM	Kucher et al	Randomized controlled study	13
	Woller et al	Retrospective cohort study (for validation)	19
MITH RAM	Zakai et al	Retrospective cohort study	20
*Bleeding RAM*			
IMPROVE Bleeding	Decousus et al	Prospective cohort study	11
	Hostler et al	Prospective cohort study (for validation)	12
	Rosenberg et al	Prospective cohort study (for validation)	16

Abbreviations: IMPROVE, International Medical Prevention Registry on Venous Thromboembolism; MITH, Medical Inpatients and Thrombosis; RAM, risk assessment model; VTE, venous thromboembolism.

More recently, in the real-world Federazione delle Associazioni dei Dirigenti Ospedalieri Internisti (FADOI)-NoTEVole (Studio nazionale, osservazionale retrospettivo, sul pattern prescrittivo della profilassi del tromboembolismo venoso in Medicina Interna alla dimissione) study, almost half of the patients admitted to IM division carried a high thrombotic score according to Padua Prediction Score, but only 7.2% presented a high bleeding risk according to IMPROVE Bleeding Score. 21 These results suggest that VTE prophylaxis can be prescribed to a large number of hospitalized, medical patients with very limited harm.

Poor evidence is available comparing the use of a RAM versus clinical judgement in choosing whether or not to prescribe VTE prophylaxis. In a single-center, prospective, quasi-randomized study, the adoption of Padua Prediction Score was associated with a 50% reduction in the incidence of VTE (either symptomatic or asymptomatic) compared with clinical judgement, with no differences in terms of bleeding and death from all cause. 12 These preliminary results need a confirmation in a large, multicenter study in order to test the appropriateness of VTE prophylaxis through a systematic application of RAMs, and to compare it with clinical judgement.

## Study Rationale and Methods

### Trial Design and Objectives

FADOI promoted the Reducing Important Clinical Outcomes in hospitalized medical ill patients (RICO) study, a multicenter, cluster-randomized, controlled clinical trial (ClinicalTrials.gov Identifier: NCT04267718) involving a large number of IM divisions affiliated to FADOI across the whole Italian territory. The aim was to evaluate the systematic use of RAMs, that is, Padua Prediction Score (PPS) and IMPROVE Bleeding Score (IBS), compared with clinical judgement as a guide for prescribing VTE prophylaxis, and the relevant clinical outcomes (thromboembolic and hemorrhagic events).

### Study Endpoints

The primary endpoint of the study is to assess the efficacy of a systematic evaluation of the thromboembolic and bleeding risk in reducing the composite rate of thromboembolic and hemorrhagic events in hospitalized, acutely ill medical patients at a 90-day follow-up.

Secondary endpoints include (i) the evaluation of clinical outcomes (VTE, major bleeding, cardiovascular events) at the time of hospital discharge (i.e., discharge at home, transfer to another unit, or death) and (ii) the assessment of VTE prophylaxis prescription during hospital stay and at the time of discharge.

As thromboprophylaxis has been shown to have the potential to reduce arterial events as well, 22 23 24 25 information about arterial events (e.g., stroke, acute myocardial infarction, peripheral artery disease) has been collected and will be analyzed as an exploratory outcome.

### Patients

#### Inclusion Criteria

Patients are included in the present study if (i) they are 18 years or older, (ii) they are hospitalized for any reason in an IM division, and (iii) they sign the informed consent.

#### Exclusion Criteria

Patients are not included in the study if (i) the expected hospital stay is less than 48 hours, (ii) they had any indication for anticoagulant therapy, or (iii) the expected life expectancy is less than 90 days.

#### Definition of the Experimental Group

This group includes those centers randomized to systematically evaluate VTE prophylaxis in all eligible patients using the Padua Prediction Score and IMPROVE Bleeding Score within 48 hours from hospitalization.

#### Definition of the Control Group

This group includes those centers randomized to evaluate VTE prophylaxis in all eligible patients based on clinical judgement only.

#### Definition of Dropout Criteria

Patients starting a de novo anticoagulant treatment during the time of hospitalization are excluded.

### Study Procedures

Centers that do not adopt any standardized procedure for the application of PPS and/or IBS are selected for the study. These centers are then randomized in a 1:1 ratio to the experimental and control arms of the study. Eligible patients in centers randomized to the experimental group are evaluated within 48 hours from hospital admission by using Padua Prediction Score and IMPROVE Bleeding Score. Centers randomized to the control group evaluate eligible patients according to clinical judgement and current clinical practice. In order to minimize any study bias, it is strongly recommended that health care personnel not involved in the patient's clinical evaluation fills the study-specific electronic case report form. All patients, irrespective of allocation arm, are assessed at a 90-day follow-up in order to evaluate the occurrence of thrombotic, hemorrhagic, or other major clinical events (deaths, cardiovascular events), and the use of VTE prophylaxis.

Study duration was originally set to 1 year. However, due to coronavirus disease 2019 pandemic, the study frame was extended to additional 2 years in order to achieve the needed sample size.

Formal approval from the Ethics Committee (EC) of the participating Centers will be obtained before any study procedure.

#### Observation and Measurements

For each enrolled patient, the following information during the hospital stay is collected: age, sex, height, and body weight (to compute body mass index), comorbidities, past medical history (with special consideration for history of VTE and recent [i.e., <3 months] history of bleeding), functional status (chronically bedridden, reduced mobility, support of a caregiver), antithrombotic and/or hormonal therapies, laboratory tests (in particular hemoglobin, platelet count, creatinine clearance), occurrence of objectively confirmed venous thromboembolic events and/or major bleeding events (according to International Society on Thrombosis and Haemostasis criteria), any other clinically relevant events, Padua Prediction Score and IMPROVE Bleeding Score (for the experimental group only), and outcome of hospitalization (discharge, transfer to another unit, death).

All patients are evaluated after 90 days through a telephone call or during an outpatient visit, if planned. The following information is collected: patient status (survival or death, in the latter case date and possible cause of death should be recorded), occurrence of objectively confirmed venous thromboembolic events and major bleeding, any other clinically relevant event, possible rehospitalization and related cause, and current antithrombotic therapies, if any. Clinical records relevant to venous thromboembolic events and major bleedings will be made available to an independent Adjudication Committee for evaluation.

#### Safety Procedures

All drugs used in this study are part of the standard-of-care according to the indications specified in the marketing authorization. The report of a suspected adverse reaction to these drugs in patients enrolled in the study is collected and reported based on current laws (D.M. April 30, 2015, as a transposition of European directives 2010/84/UE e 2012/26/UE) by filling a report form of suspected adverse reaction within the time specified by the law.

The report form can be filled electronically by accessing the platform Vigifarmaco ( www.vigifarmaco.it ) through the guided procedure, manually by paper or by fax or e-mail to the Local Pharmacovigilance Manager of the hospital. In addition, a copy of the form must be emailed or faxed to the study promoter.

### Ethical Considerations

This study is conducted in compliance with the protocol and in accordance with Centro Studi Fondazione FADOI standard operating procedures. These are designed to ensure adherence to Good Clinical Practice, International Conference on Harmonization Harmonized Tripartite Guidelines for Good Clinical Practice and Declaration of Helsinki. 26

#### Institutional Review Board/Ethics Committee

Before implementing this study, the protocol, the proposed informed consent form, and any other information relevant to enrolled subjects were reviewed by the Institutional Review Board (IRB)/EC of the participating centers. A signed and dated statement confirming that the protocol and the informed consent were approved by the IRB/EC of the participating center was sent to Centro Studi Fondazione FADOI before study initiation.

#### Informed Consent

The investigator must explain to each subject (or legally authorized representative) the nature of the study, its purpose, the procedures, the expected duration, the potential risks and benefits involved, and any discomfort it may entail. Each subject must be informed that participation in the study is voluntary and that he/she may withdraw his/her consent for the study at any time. Consent withdrawal will not affect his/her subsequent treatment or relationship with the attending physician. This informed consent is given by means of a standard written statement in nontechnical language. The subject should read and consider the statement before signing and dating, and should be given a copy of the signed document. If written consent is not possible, oral consent can be obtained if witnessed by a signed statement from one or more people not involved in the study. It should also be mentioned why the patient was unable to sign the form. No subject can enter the study before his/her informed consent has been obtained.

### Statistical Considerations

#### Sample size

In order to demonstrate a 50% reduction in the proportion of the primary outcome in the experimental group and assuming an incidence of the primary outcome of 3.5% in the control group at 90 days, 16 clusters of at least 90 patients for each group are needed to have a fixed number of equal-sized randomized clusters, with 80% power at a 5% significance level (two-sided) and with an intracluster correlation coefficient of 0.001. Considering a potential dropout rate of 5%, at least 32 centers for a total of 2,844 patients are required for the study.

#### Statistical Analysis

Data will be analyzed and reported using the Consolidated Standard of Reporting Trials and the International Conference on Harmonisation E9 guidelines. All primary analyses will be on an intention-to-treat basis including all randomized participants.

Baseline characteristics, collected at the time of enrollment, will be cross-tabulated, according to the randomized group to check for appropriate balance and provide an overview of the study population. Baseline characteristics will be summarized as mean and standard deviation for continuous variables and as frequencies and percentages for categorical ones.

The primary outcome (proportion of thromboembolic and hemorrhagic events at a 90-d follow-up after hospital discharge) will be analyzed through an univariate logistic regression analysis to check for variables differing between groups. Variables significant at *p* ≤ 0.10 will be included as covariates in the multilevel logistic regression model that takes into account the clustered nature of the data. As RICO is a randomized trial, we do not have a priori potential confounders. A propensity score analysis as an additional sensitivity analysis will be also performed. Considering the potential risk of an unbalanced recruitment across participating centers, the use of a multilevel logistic regression model is solid in case of unequal cluster size. 27 Secondary outcomes will be compared between groups and follow the approach detailed above for primary outcome.

Numbers and percentages of adverse events (AEs) and serious AEs will be cross-tabulated with allocated group. According to the study protocol and the primary outcome of the study, thromboembolic and hemorrhagic events will not be considered AEs.

## Discussion

As VTE still represents an important cause of morbidity and mortality among hospitalized, medically ill patients, efforts are warranted to reduce this burden. Thromboprophylaxis is underprescribed 1 and this might depend on the difficult balance between prothrombotic and bleeding risk in medical patients. Indeed, these patients are characterized by a number of comorbidities and related pharmacological treatments with possible drug-to-drug interactions. Contemporary guidelines recommend a systematic use of RAMs including both VTE and bleeding risk to decide whether to start VTE prophylaxis. 4 However, these recommendations are based on relatively weak evidence. Currently available RAMs evaluating VTE risk in hospitalized patients derive from observational studies, as depicted in Table 1 . Although both Padua Prediction Score and IMPROVE Bleeding Score have been validated in a number of cohort studies, an impact analysis assessed through a randomized controlled trial is eagerly awaited in order to implement RAMs in clinical practice. Unfortunately, this analysis is currently lacking for Padua Prediction Score and IMPROVE Bleeding Score.

Attempts to overcome the problem of thromboprophylaxis under prescription were undertaken in the past years. In a randomized, controlled study of >2,000 hospitalized patients, the use of a strategy including an electronic alert about VTE risk versus no alert greatly reduced the occurrence of symptomatic VTE (deep vein thrombosis or pulmonary embolism) at 90 days (hazard ratio [HR] 0.59, 95% confidence interval [CI], 0.43–0.81; *p* = 0.001) with no increase in the bleeding rate. 13 A randomized clinical trial testing the effectiveness of an alert from a hospital staff member to the attending physician for VTE prophylaxis demonstrated to reduce the rate of symptomatic VTE at 90 days without reaching statistical significance (HR 0.79, 95% CI 0.50–1.25) with no increase in bleeding events at 30 days. 28 A similar strategy was tested in patients needing extended VTE prophylaxis after hospital discharge, however, no decrease in the rate of symptomatic VTE was observed. 29 The generally positive influence provided by an intervention aimed at increasing VTE prophylaxis prescription was documented by other studies with disappointing results on VTE incidence reduction. 30 31

These studies did not consider the systematic use of RAMs, although they included differently weighted risk factors for VTE. In a single-center, quasi-randomized trial, the adoption of Padua Prediction Score versus clinical judgement in medical patients was compared. 12 This study showed a 50% reduction in the incidence of VTE in those with Padua Prediction Score-guided thromboprophylaxis, with no difference in terms of bleeding or death from all causes. 12 In another single-center, retrospective, and prospective observational study, the incidence of venous thromboembolic and hemorrhagic events in consecutive patients admitted to an IM department before and after the introduction and extensive use of RAMs was compared (203 patients in the retrospective group and 210 patients in the prospective group, respectively). 13 Despite a statistically significant decrease in pharmacological VTE prophylaxis after implementation of RAMs (43.3 vs. 56.7%, *p* = 0.028), the incidence of VTE was not affected, suggesting that RAM introduction may be used to safely reduce health expenditure associated with VTE prophylaxis in hospitalized medical patients.

Considering this evidence, the RICO trial will first aim to confirm previous findings about the importance of VTE prophylaxis. Therefore, the main objective of the trial is the systematic assessment of the thrombotic and bleeding risk in order to reduce thromboembolic and hemorrhagic events at 90 days. This latter aspect is essential when compared to the recently completed trial by the IMPROVE group (A Universal Electronic Health Record-based IMPROVE VTE Risk Assessment Model for the Prevention of Venous Thromboembolism in Hospitalized Medically Ill Patients, NCT04768036). Indeed, this trial will not address as a primary outcome the occurrence of VTE-related complications at 90 days, as we will do in the RICO trial. More importantly, the IMPROVE trial will not consider using RAMs to allocate patients to the treatment group—as we will do in the RICO trial—in favor of a computed platform (“SMART on FHIR” platform-based Electronic Health Record (EHR)-embedded IMPROVE DD VTE clinical prediction rules with electronic order entry). Finally, the RICO trial will include younger patients than the IMPROVE trial (age > 60 years is an eligibility criterion).

RAMs are not routinely included in clinical decision-making, which remains based on clinical judgement. 14 The latter, however, has a limited support from the available literature. In addition, it should be underlined that in-hospital VTE and/or hospital readmission for VTE represent an important medical cost for the health care system. 32 33

Given the scarcity of randomized trials assessing the benefit of RAM- versus clinical judgement-guided VTE prophylaxis, the RICO study aims to evaluate which approach might reduce the composite, long-term incidence of these complications in hospitalized, acutely ill medical patients during hospital stay and after discharge. The results of this trial are expected to increase the knowledge on this topic. In particular, whether a RAM-guided strategy will result in improved outcomes, the study will contribute to strengthen the systematic use of Padua Prediction Score and IMPROVE Bleeding Score in the routine clinical practice.

## References

[1] GEMINI Study Group Gussoni G Campanini M Silingardi M In-hospital symptomatic venous thromboembolism and antithrombotic prophylaxis in Internal Medicine. Findings from a multicenter, prospective study Thromb Haemost 2009 101 05 893 901 19404543

[2] Heit J A Epidemiology of venous thromboembolism Nat Rev Cardiol 2015 12 08 464 474 26076949 10.1038/nrcardio.2015.83 PMC4624298

[3] Dentali F Douketis J D Gianni M Lim W Crowther M A Meta-analysis: anticoagulant prophylaxis to prevent symptomatic venous thromboembolism in hospitalized medical patients Ann Intern Med 2007 146 04 278 288 17310052 10.7326/0003-4819-146-4-200702200-00007

[4] Schünemann H J Cushman M Burnett A E American Society of Hematology 2018 guidelines for management of venous thromboembolism: prophylaxis for hospitalized and nonhospitalized medical patients Blood Adv 2018 2 22 3198 3225 30482763 10.1182/bloodadvances.2018022954 PMC6258910

[5] Cave B Hough A Dobesh P P Extended venous thromboembolism prophylaxis in medically ill patients Pharmacotherapy 2018 38 06 597 609 29543384 10.1002/phar.2102

[6] Mahan C E Fisher M D Mills R M Thromboprophylaxis patterns, risk factors, and outcomes of care in the medically ill patient population Thromb Res 2013 132 05 520 526 24080150 10.1016/j.thromres.2013.08.013

[7] TERSICORE Study Group Scannapieco G Ageno W Airoldi A Incidence and predictors of venous thromboembolism in post-acute care patients. A prospective cohort study Thromb Haemost 2010 104 04 734 740 20664897 10.1160/TH10-03-0169

[8] Barbar S Noventa F Rossetto V A risk assessment model for the identification of hospitalized medical patients at risk for venous thromboembolism: the Padua Prediction Score J Thromb Haemost 2010 8 11 2450 2457 20738765 10.1111/j.1538-7836.2010.04044.x

[9] Blondon M Righini M Nendaz M External validation of the simplified Geneva risk assessment model for hospital-associated venous thromboembolism in the Padua cohort J Thromb Haemost 2020 18 03 676 680 31782886 10.1111/jth.14688

[10] Chopard P Spirk D Bounameaux H Identifying acutely ill medical patients requiring thromboprophylaxis J Thromb Haemost 2006 4 04 915 916 16634771 10.1111/j.1538-7836.2006.01818.x

[11] IMPROVE Investigators Decousus H Tapson V F Bergmann J F Factors at admission associated with bleeding risk in medical patients: findings from the IMPROVE investigators Chest 2011 139 01 69 79 20453069 10.1378/chest.09-3081

[12] Hostler D C Marx E S Moores L K Validation of the International Medical Prevention Registry on Venous Thromboembolism Bleeding Risk Score Chest 2016 149 02 372 379 26867833 10.1378/chest.14-2842

[13] Kucher N Koo S Quiroz R Electronic alerts to prevent venous thromboembolism among hospitalized patients N Engl J Med 2005 352 10 969 977 15758007 10.1056/NEJMoa041533

[14] Nendaz M Spirk D Kucher N Multicentre validation of the Geneva Risk Score for hospitalised medical patients at risk of venous thromboembolism. Explicit ASsessment of Thromboembolic RIsk and Prophylaxis for Medical PATients in SwitzErland (ESTIMATE) Thromb Haemost 2014 111 03 531 538 24226257 10.1160/TH13-05-0427

[15] Rosenberg D Eichorn A Alarcon M McCullagh L McGinn T Spyropoulos A C External validation of the risk assessment model of the International Medical Prevention Registry on Venous Thromboembolism (IMPROVE) for medical patients in a tertiary health system J Am Heart Assoc 2014 3 06 e001152 25404191 10.1161/JAHA.114.001152 PMC4338701

[16] Rosenberg D J Press A Fishbein J External validation of the IMPROVE Bleeding Risk Assessment Model in medical patients Thromb Haemost 2016 116 03 530 536 27307054 10.1160/TH16-01-0003

[17] IMPROVE Investigators Spyropoulos A C Anderson F A Jr FitzGerald G Predictive and associative models to identify hospitalized medical patients at risk for VTE Chest 2011 140 03 706 714 21436241 10.1378/chest.10-1944

[18] Vardi M Ghanem-Zoubi N O Zidan R Yurin V Bitterman H Venous thromboembolism and the utility of the Padua Prediction Score in patients with sepsis admitted to internal medicine departments J Thromb Haemost 2013 11 03 467 473 23279085 10.1111/jth.12108

[19] Woller S C Stevens S M Jones J P Derivation and validation of a simple model to identify venous thromboembolism risk in medical patients Am J Med 2011 124 10 947 95400 21962315 10.1016/j.amjmed.2011.06.004

[20] Zakai N A Callas P W Repp A B Cushman M Venous thrombosis risk assessment in medical inpatients: the medical inpatients and thrombosis (MITH) study J Thromb Haemost 2013 11 04 634 641 23336744 10.1111/jth.12147

[21] FADOI-NoTEVole Study Group Squizzato A Agnelli G Campanini M Prophylaxis of venous thromboembolism after hospital discharge in internal medicine: findings from the observational FADOI-NoTEVole study Thromb Haemost 2019 119 12 2043 2052 31634959 10.1055/s-0039-1697661

[22] APEX Investigators Gibson C M Chi G Halaby R Extended-duration betrixaban reduces the risk of stroke versus standard-dose enoxaparin among hospitalized medically ill patients: an APEX Trial Substudy (Acute Medically Ill Venous Thromboembolism Prevention With Extended Duration Betrixaban) Circulation 2017 135 07 648 655 27881569 10.1161/CIRCULATIONAHA.116.025427

[23] APEX Investigators Gibson C M Korjian S Chi G Comparison of fatal or irreversible events with extended-duration betrixaban versus standard dose enoxaparin in acutely ill medical patients: an APEX Trial Substudy J Am Heart Assoc 2017 6 07 e006015 28698258 10.1161/JAHA.117.006015 PMC5586307

[24] Raskob G E Spyropoulos A C Cohen A T Association between asymptomatic proximal deep vein thrombosis and mortality in acutely ill medical patients J Am Heart Assoc 2021 10 05 e019459 33586478 10.1161/JAHA.120.019459 PMC8174250

[25] Spyropoulos A C Ageno W Albers G W Post-discharge prophylaxis with rivaroxaban reduces fatal and major thromboembolic events in medically ill patients J Am Coll Cardiol 2020 75 25 3140 3147 32586587 10.1016/j.jacc.2020.04.071 PMC7308003

[26] International Conference on Harmonisation of technical requirements for registration of pharmaceuticals for human use. ICH harmonized tripartite guideline: Guideline for Good Clinical Practice J Postgrad Med 2001 47 01 45 50 11590294

[27] Heo M Leon A C Performance of a mixed effects logistic regression model for binary outcomes with unequal cluster size J Biopharm Stat 2005 15 03 513 526 15920895 10.1081/BIP-200056554

[28] Piazza G Rosenbaum E J Pendergast W Physician alerts to prevent symptomatic venous thromboembolism in hospitalized patients Circulation 2009 119 16 2196 2201 19364975 10.1161/CIRCULATIONAHA.108.841197 PMC2901546

[29] Piazza G Anderson F A Ortel T L Randomized trial of physician alerts for thromboprophylaxis after discharge Am J Med 2013 126 05 435 442 23510945 10.1016/j.amjmed.2012.09.020

[30] Lecumberri R Marqués M Díaz-Navarlaz M T Maintained effectiveness of an electronic alert system to prevent venous thromboembolism among hospitalized patients Thromb Haemost 2008 100 04 699 704 18841295 10.1160/th08-05-0337

[31] Baroletti S Munz K Sonis J Electronic alerts for hospitalized high-VTE risk patients not receiving prophylaxis: a cohort study J Thromb Thrombolysis 2008 25 02 146 150 18026689 10.1007/s11239-007-0081-1

[32] Spyropoulos A C Lin J Direct medical costs of venous thromboembolism and subsequent hospital readmission rates: an administrative claims analysis from 30 managed care organizations J Manag Care Pharm 2007 13 06 475 486 17672809 10.18553/jmcp.2007.13.6.475 PMC10437642

[33] Tatar M Senturk A Tuna E Local cost study of treatment of venous thromboembolism in Turkey Value Health 2015 18 07 A388 A389

[34] Mahan C E Liu Y Turpie A G External validation of a risk assessment model for venous thromboembolism in the hospitalised acutely-ill medical patient (VTE-VALOURR) Thromb Haemost 2014 112 04 692 699 24990708 10.1160/TH14-03-0239

[35] Greene M T Spyropoulos A C Chopra V Validation of risk assessment models of venous thromboembolism in hospitalized medical patients Am J Med 2016 129 09 1.001E12 1.001E21 27107925 10.1016/j.amjmed.2016.03.031

